# Synthesis of Novel 3,5-Dichloro-2-arylpyridines by Palladium Acetate-Catalyzed Ligand-Free Suzuki Reactions in Aqueous Media

**DOI:** 10.3390/molecules14093153

**Published:** 2009-08-26

**Authors:** Huanan Hu, Changhua Ge, Anjiang Zhang, Lisheng Ding

**Affiliations:** 1 College of Pharmaceutial and Chemical Engineering, Taizhou University, Linhai 317000, China; 2 Ningbo Institute of Material Technology and Engineering, Chinese Academy of Sciences, Ningbo 315201, China; 3 Chengdu Institute of Biology, Chinese Academy of Sciences, Chengdu 610041, China

**Keywords:** Suzuki reaction, ligand-free, aqueous phase, 2,3,5-trichloropyridine, 3,5-dichloro-2-arylpyridines

## Abstract

A highly efficient palladium acetate-catalyzed ligand-free Suzuki reaction of 2,3,5-trichloropyridine with arylboronic acids in aqueous phase was developed. High yields of 3,5-dichloro-2-arylpyridines, a simple Pd source, absence of ligands, and environmentally benign as well as mild reaction conditions are important features of this method.

## Introduction

Heterocyclic compounds used as medicines and pesticides have developed rapidly in recent years and have become the new trend [1]. Both natural and artificial heterocyclic compounds are all very important in medicine and pesticide research. There are numerous examples in the literature of the biological activity of biaryls with pyridine rings, which are used in pharmaceuticals [2,3,4,5], herbicides [6,7,8] and even light emitting materials [9,10,11,12,13,14]. Palladium-catalyzed cross-coupling of aryl halides with organoboronic acids, known as the Suzuki cross-coupling reaction, is a versatile and highly utilized reaction for the selective formation of carbon–carbon bonds, in particular for the synthesis of biaryls [2,3,4,5]. Recently, efforts have been focused on the development of efficient and selective catalytic systems for the Suzuki reaction, but many such catalytic systems are limited to the couplings of aromatic iodides and bromides because of the much higher energy is required for the oxidative insertion of palladium catalysts into the C-Cl bond of aryl chlorides [5,14,15,16,17,18,19,20]. In recent years, the use of readily available aryl chlorides in these transformations has received increasing attention, and a number of effective catalytic systems have been developed for this purpose [18,19,20]. In these processes, the use of sterically hindered and electron-rich ligands played crucial roles in the coupling of these challenging substrates. However, the reaction carried out in conventional organic solvents suffers from several drawbacks including high catalyst consumption, high reaction temperatures and decomposition of catalyst, which place significant limits on their synthetic applications.

There have been few reports on palladium catalyzed ligand-free Suzuki coupling of heteroaromatic chloride with arylboronic acids in aqueous phase. 2,3,5-Trichloropyridine can be used as intermediate for the production of active herbicidal substances [21,22]. Herein, we presented a palladium acetate-catalyzed ligand-free Suzuki coupling of 2,3,5-trichloropyridine with arylboronic acids in aqueous phase, and its use in the regioselective synthesis of a series of novel 3,5-dichloro-2-arylpyridines. Of interest was the fact that no di- or tri-substituted products were obtained in these reactions.

## Results and Discussion

**Scheme 1:**
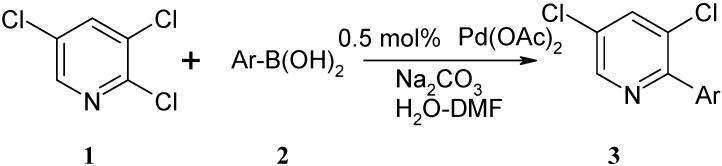
Suzuki coupling of 2,3,5-trichloropyridine with arylboronic acids.

Wallow and co-workers [23] have reported an improved method for ligandless Suzuki couplings using 1:1 acetone/water as solvent. However, the attractiveness of their protocol is limited by the painstaking measures used to exclude oxygen, including a total of 15 freeze-pump-thaw degassing cycles at various stages of the reaction. Liu and co-workers [24] have reported that a highly efficient palladium acetate-catalyzed ligand-free Suzuki reaction of aryl iodides and bromides with arylboronic acids in aqueous phase took place with short reaction times (0.5-1 h) at 35 °C in air. The key for such a successful catalytic system was the use of a suitable amount of an organic cosolvent, with the best ratio being a 3.5:3 mixture of water and organic solvent. We have used conditions similar to those described by Liu to study a ligand-free Suzuki coupling of 2,3,5-trichloropyridine with arylboronic acids in aqueous phase (Scheme 1).

For the investigation of reaction conditions for the ligand-free Suzuki coupling of 2,3,5-trichloropyridine with arylboronic acids in aqueous phase, we chose the reaction of 2,3,5-trichloropyridine with phenylboronic acid as a model. We initially studied the effect of a solvent on the yields. The reactions were carried out using Na_2_CO_3_ as base in a 3.5:3 mixture of water and organic solvent in the presence of 0.5 mol % Pd(OAc)_2_ in air. We found the use of ethanol, propanol, acetone and DMF as cosolvents all gave good yields (Table 1, entries 1-4), and DMF was found to be the best solvent, whereas toluene, CH_2_Cl_2_ and THF provided very low yields (Table 1, entries 5-7). We found that the best yield of **3a** was obtained when the reaction temperature was increased to 60 °C in DMF/H_2_O (3:3.5 mL) (Table 1, entries 4, entries 8). However, reaction yields decreased as the reaction temperature was increased. The results are shown in Table 1.

We also surveyed the effect of reaction time on the yields using DMF as cosolvent. The best yields could be obtained after 12 h at 60 °C, and the reaction yields decreased for more than or less than 12 h. The results are given in Table 2.

Under the optimized reaction conditions [Pd(OAc)_2_ as catalyst, Na_2_CO_3_ as base and H_2_O/DMF as cosolvent], a series of novel 3,5-dichloro-2-arylpyridines were regioselectively synthesized by palladium acetate-catalyzed ligand-free Suzuki reaction in aqueous phase, and results are summarized in Table 3.

Aryl boronic acids with electron withdrawing substituted groups, which are less nucleophilic and, hence, transmetalate more slowly than electron-neutral analogues, are prone to homocoupling and protodeboronation side reactions [25]. However, in our system, both the electron rich and the electron-deficient arylboronic acids afforded the corresponding products in good yields.

Because much higher energy is required for the oxidative insertion of palladium catalysts into the C-Cl bond of aryl chlorides, transformations of these substrates remain a significant challenge in organic synthesis. We have regioselectively synthesized a series of 3,5-dichloro-2-arylpyridines, and no di- or tri-substituted products were found in these reactions, so the reaction of different substituted chloropyridines with arylboronic acid were studied, and the results were summarized in Table 4.

We found 2-chloropyridine, which is electron deficient, afforded the products in 76% yield, while 3-chloropyridine was almost inactive. We also found 2,3,5-trichloropyridine gave better yields as compared to 2-chloropyridine, we conjectured the activity of 2-position chlorine in 2,3,5-trichloropyridine was enhanced because of the withdrawing action on ortho nitrogen and 3,5-position chlorine, hence, 3,5-dichloro-2-arylpyridines were regioselectively synthesized in mild reaction conditions.

## Conclusions

In conclusion, a highly efficient and environmentally friendly protocol to synthesis of novel 3,5-dichloro-2-arylpyridines has been developed. Important features of present method include a simple Pd source, no need for ligands, environmentally benign and mild reaction conditions and good yields. Further studies on the biological activity of 3,5-dichloro-2-arylpyridines are in progress.

## Experimental

### General

All melting points were determined on an XT-4A apparatus and are uncorrected. TLC was performed using precoated silica gel GF_254_ (0.25 mm), column chromatography was performed using silica gel (200-300 mesh). The ^1^H- and ^13^C-NMR spectra were measured at 25 °C at 300 and 75 MHz, respectively, on a Bruker Advance 300 spectrometer, using TMS as internal standard. *J*-values are given in Hz. The IR spectra were taken on a Bruker Vector 55 spectrometer. Elemental analyses were carried out with an EA 1112 elemental analyzer. 2,3,5-Trichloropyridine (**1**) was prepared according to the reported procedure [21]. Aryl bromides were used directly as obtained commercially unless otherwise noted.

### Typical procedure for the preparation of 3,5-dichloro-2-arylpyridines ***3***

A mixture of Na_2_CO_3_ (0.212 g, 2 mmol), Pd(OAc)_2_ (1 mg, 0.5 mol %), 2,3,5-trichloropyridine (1 mmol), arylboronic acid (1.5 mmol), distilled water (3.5 mL) and DMF (3 mL) was stirred at 60 °C for 12 h. Afterward, the reaction solution was extracted four times with diethyl ether (4 × 10 mL). The combined organic extract was concentrated under reduced pressure and the residue was subjected to chromatography on a column of silica gel, eluting with petroleum ether and ethyl acetate. The eluate was cocentrated under reduced pressure, giving compounds **3a–j**.

*3,5-Dichloro-2-phenylpyridine* (**3a**): Colorless solid; m.p. 38-39 °C; ^1^H-NMR (CDCl_3_) *δ*: 8.55 (d, *J* = 2.1 Hz, 1H), 7.77 (d, *J* = 2.1 Hz, 1H), 7.70 (m, 2H), 7.48 (m, 2H), 7.33 (m, 1H); ^13^C-NMR (CDCl_3_) *δ*: 154.7, 146.5, 137.4, 133.8, 130.4, 130.1, 129.2, 128.5, 128.1; IR (KBr) ν: 3057, 1616, 1565, 1430, 1370, 1199, 1115 cm^-1^; Anal. Calcd. for C_11_H_7_Cl_2_N: C, 58.96; H, 3.15; N, 6.25. Found: C, 59.12; H, 3.18; N, 6.19%.

*3,5-Dichloro-2-p-tolylpyridine* (**3b**): Colorless solid; m.p. 51-52 °C; ^1^H-NMR (CDCl_3_) *δ*: 8.53 (d, *J* = 2.1 Hz, 1H), 7.80 (d, *J* = 2.1 Hz, 1H), 7.61 (d, *J* = 8.2 Hz, 2H), 7.30 (m, 1H), 2.41 (s, 3H); ^13^C-NMR (CDCl_3_) *δ*: 146.4, 139.1, 137.3, 134.4, 133.8, 130.1, 129.2, 128.8, 128.4, 21.3; IR (KBr) ν: 3053, 2918, 1612, 1565, 1431, 1372, 1195, 1111 cm^-1^; Anal. Calcd. for C_12_H_9_Cl_2_N: C, 60.53; H, 3.81; N, 5.88. Found: C, 60.45; H, 3.86; N, 5.94%.

*3,5-Dichloro-2-o-tolylpyridine* (**3c**): Yellowish oil; ^1^H-NMR (CDCl_3_) *δ*: 8.55 (d, *J* = 2.1 Hz, 1H), 7.83 (d, *J* = 2.1 Hz, 1H), 7.28 (m, 4H), 2.15 (s, 3H); ^13^C-NMR (CDCl_3_) *δ*: 156.2, 146.3, 137.1, 136.6, 135.9, 131.4, 130.7, 130.2, 128.9, 128.8, 125.7, 19.4; IR (film) ν: 3055, 2923, 1649, 1533, 1432, 1368, 1196, 1114 cm^-1^; Anal. Calcd. for C_12_H_9_Cl_2_N: C, 60.53; H, 3.81; N, 5.88. Found: C, 60.63; H, 3.86; N, 5.79%.

*3,5-Dichloro-2-(4-chlorophenyl)pyridine* (**3d**): Colorless solid; m.p. 126-127 °C; ^1^H-NMR (CDCl_3_) *δ*: 8.54 (d, *J* = 2.0 Hz, 1H), 7.82 (d, *J* = 2.0 Hz, 1H), 7.66 (d, *J* = 8.4 Hz, 2H), 7.38 (m, 2H); ^13^C-NMR (CDCl_3_) *δ*: 1543.4, 146.6, 143.4, 137.5, 135.4, 130.7, 130.0, 128.4, 122.7; IR (KBr) ν: 3065, 1595, 1564, 1488, 1435, 1364, 1199, 1115 cm^-1^; Anal. Calcd. for C_11_H_6_Cl_3_N: C, 51.10; H, 2.34; N, 5.42. Found: C, 50.97; H, 2.35; N, 5.48%.

*3,5-Dichloro-2-(4-fluorophenyl)pyridine* (**3e**): Colorless solid; m.p. 119-121 °C; ^1^H-NMR (CDCl_3_) *δ*: 8.53 (d, *J* = 2.1 Hz, 1H), 7.82 (d, *J* = 2.1 Hz, 1H), 7.71 (m, 2H), 7.15 (m, 2H); ^13^C-NMR (CDCl_3_) *δ*: 164.8, 161.5, 153.6, 146.5, 137.5, 133.1, 131.3(q, *J* = 8.3 Hz, 1C), 130.5, 115.2(q, *J* = 21.7 Hz, 1C); IR (KBr) ν: 3046, 1602, 1565, 1508, 1437, 1367, 1166, 1119 cm^-1^; Anal. Calcd. for C_11_H_6_Cl_2_FN: C, 54.58; H, 2.50; N, 5.79. Found: C, 54.19; H, 2.53; N, 5.86%.

*3,5-Dichloro-2-(4-methoxyphenyl)pyridine* (**3f**): Colorless solid; m.p. 86-88 °C; ^1^H-NMR (CDCl_3_) *δ*: 8.51 (d, *J* = 2.1 Hz, 1H), 7.77 (d, *J* = 2.1 Hz, 1H), 7.70 (d, *J* = 8.8 Hz, 2H), 6.98 (d, *J* = 8.8 Hz, 2H), 3.84 (s, 3H); ^13^C-NMR (CDCl_3_) *δ*: 160.3, 154.2, 146.4, 137.4, 130.8, 129.8, 129.7, 129.5, 113.5, 55.3; IR (film) ν: 3048, 2926, 1606, 1509, 1434, 1366, 1175, 1113 cm^-1^; Anal. Calcd. for C_12_H_9_Cl_2_NO: C, 56.72; H, 3.57; N, 5.51. Found: C, 56.66; H, 3.60; N, 5.47%.

*3,5-Dichloro-2-(3-(trifluoromethyl)phenyl)pyridine* (**3g**): Yellowish oil; ^1^H-NMR (CDCl_3_) *δ*: 8.57 (d, *J* = 2.1 Hz, 1H), 7.92 (d, *J* = 2.1 Hz, 1H), 7.79-7.85 (m, 2H), 7.56-7.71 (m, 2H); ^13^C-NMR (CDCl_3_) *δ*: 153.0, 146.7, 143.4, 139.0, 137.7, 132.6, 131.2 (q, *J* = 34.5 Hz, 1C), 128.6, 126.5, 125.8, 123.8 (q, *J* = 271.3 Hz, 1C); IR (film) ν: 3067, 2923, 1616, 1565, 1490, 1425, 1373, 1169, 1128 cm^-1^; Anal. Calcd. for C_12_H_6_Cl_2_F_3_N: C, 49.34; H, 2.07; N, 4.80. Found: C, 49.41; H, 2.10; N, 4.71%.

*3,5-Dichloro-2-(3-nitrophenyl)pyridine* (**3h**): Colorless solid; m.p. 107-108 °C; ^1^H-NMR (CDCl_3_) *δ*: 8.64 (d, *J* = 1.6 Hz,, 1H), 8.59 (d, *J* = 1.6 Hz, 1H), 8.27-8.31 (m, 1H), 8.23 (d, *J* = 8.0 Hz, 1H), 7.88 (d, *J* = 1.6 Hz, 1H), 7.55-7.80 (m, 1H); ^13^C-NMR (CDCl_3_) *δ*: 151.9, 146.9, 146.0, 138.5, 137.7, 135.3, 131.7, 130.2, 129.2, 124.5, 123.4; IR (KBr) ν: 3056, 1617, 1565, 1485, 1431, 1348, 1118 cm^-1^; Anal. Calcd. for C_11_H_6_Cl_2_N_2_O_2_: C, 49.10; H, 2.25; N, 10.41. Found: C, 49.23; H, 2.25; N, 10.39%.

*3,5-Dichloro-2-(thiophen-3-yl)pyridine* (**3i**): Colorless solid; m.p. 42-43 °C; ^1^H-NMR (CDCl_3_) *δ*: 8.49 (d, *J* = 2.1 Hz, 1H), 8.02 (d, *J* = 2.1 Hz, 1H), 7.68-7.78 (m, 1H), 7.30-7.38 (m, 2H); ^13^C-NMR (CDCl_3_) *δ*: 149.4, 146.3, 137.6, 133.7, 129.6, 128.6, 128.1, 127.2, 125.0; IR (KBr) ν: 3048, 1615, 1562, 1432, 1373, 1195, 1111 cm^-1^; Anal. Calcd. for C_9_H_5_Cl_2_NS: C, 46.98; H, 2.19; N, 6.09. Found: C, 47.06; H, 2.17; N, 6.14%.

*3,5-Dichloro-2-(naphthalen-1-yl)pyridine* (**3j**): Colorless solid; m.p. 63-64 °C; ^1^H-NMR (CDCl_3_) *δ*: 8.65 (d, *J* = 2.0 Hz, 1H), 7.91-7.99 (m, 3H), 7.70 (m, 2H), 7.44-7.57 (m, 5H); ^13^C-NMR (CDCl_3_) *δ*: 155.2, 146.5, 136.9, 134.9, 133.5, 132.2, 131.1, 130.9, 129.4, 128.5, 127.2, 126.5, 126.0, 125.1, 124.9; IR (KBr) ν: 3056, 1612, 1566, 1454, 1380, 1189, 1115 cm^-1^; Anal. Calcd. for C_15_H_9_Cl_2_N: C, 65.72; H, 3.31; N, 5.11. Found: C, 65.81; H, 3.35; N, 5.01%.

### Typical Procedure for the preparation of 2-phenylpyridine *(**4**)*

A mixture of Na_2_CO_3_ (0.212 g, 2 mmol), Pd(OAc)_2_ (1 mg, 0.5 mol %), 2-chloropyridine (1 mmol), phenylboronic acid (1.5 mmol), distilled water (3.5 mL) and DMF (3 mL) was stirred at 60 °C for 12 h. Afterward, the reaction solution was extracted four times with diethyl ether (4×10 mL). The organic solvent was removed under reduced pressure. The residue was subjected to chromatography on a column of silica gel, eluting with petroleum ether and ethyl acetate, and the solvent was removed under reduced pressure, giving compound **4**. Yellowish oil; ^1^H-NMR (CDCl_3_) *δ*: 8.59 (d, *J* = 4.8 Hz, 1H), 7.99 (d, *J* = 6.8 Hz, 2H), 7.78–7.71 (m, 2H), 7.48 (t, J = 8.8Hz, 2H), 7.42 (t, J = 7.2Hz, 1H), 7.26–7.21 (m, 1H); ^13^C-NMR (CDCl_3_) *δ*: 157.4, 149.6, 139.3, 136.7, 128.9, 128.7, 126.9, 122.1, 120.5; IR (film) ν: 3056, 1610, 1565, 1450, 1316, 1182, 1095 cm^-1^.

## Figures and Tables

**Table 1:** Effect of solvent on the Suzuki reaction *^a^*.

Entry	Solvent (3:3.5 mL)	Yield*^b^* (%)
1	acetone/H_2_O	81
2	ethanol/H_2_O	80
3	propanol/H_2_O	79
4	DMF/H_2_O	83
5	DCM/H_2_O	29
6	THF/H_2_O	34
7	Toluene/H_2_O	21
8*^c^*	DMF/H_2_O	89

*^a^* Reaction conditions: 2,3,5-trichloropyridine (1 mmol), phenylboronic acid (1.5 mmol), Na_2_CO_3_ (2 mmol), Pd(OAc)_2_ (0.5% mmol), H_2_O/solvent =3.5:3 mL, at 35 °C, for 12 h; *^b^* Isolated yields; *^c^* Reaction time at 60 °C.

**Table 2:** Effect of reaction time on the Suzuki reaction *^a^*.

**Time/h**	4	6	8	10	12	14
**Yield*^b^*/%**	55	61	79	85	89	87

*^a^* Reaction conditions: 2,3,5-trichloropyridine (1 mmol), phenylboronic acid (1.5 mmol), Na_2_CO_3_ (2 mmol), Pd(OAc)_2_ (0.5 % mmol), H_2_O/DMF =3.5:3 mL, at 60°C; *^b^* Isolated yields.

**Table 3:** Suzuki coupling of 2,3,5-trichloropyridine with arylboronic acids *^a^*.

Entry	Arylboronic acid	Product(3)	Yield*^b^* (%)
1	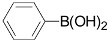	**3a**	89
2	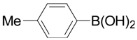	**3b**	90
3	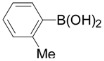	**3c**	87
4	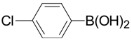	**3d**	91
5	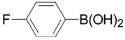	**3e**	89
6	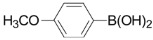	**3f**	85
7	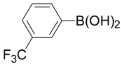	**3g**	88
8	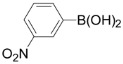	**3h**	82
9	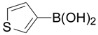	**3i**	86
10	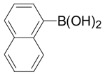	**3j**	89

*^a^* Reaction conditions: 2,3,5-trichloropyridine (1 mmol), arylboronic acid (1.5 mmol), Na_2_CO_3_ (2 mmol), Pd(OAc)_2_ (0.5 % mmol), H_2_O/DMF = 3.5:3 mL, at 60°C, for 12 h; *^b^* Isolated yields.

**Table 4:** Suzuki coupling of different chloropyridines with phenylboronic acids *^a^*.

Entry	chloropyridine	phenylboronic acid	Product	Yield*^b^*(%)
1	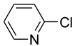	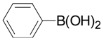	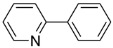 **4**	76
2	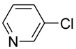	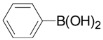	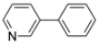 **5**	trace
3	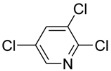	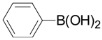	**3a**	89

*^a^* Reaction conditions: chloropyridine (1 mmol), phenylboronic acid (1.5 mmol), Na_2_CO_3_ (2 mmol), H_2_O/DMF = 3.5:3 mL, at 60 °C, for 12 h; *^b^* Isolated yields.
